# Mechanical Properties Analysis of the AA2519-AA1050-Ti6Al4V Explosive Welded Laminate

**DOI:** 10.3390/ma13194348

**Published:** 2020-09-30

**Authors:** Ireneusz Szachogluchowicz, Lucjan Sniezek, Tomasz Slezak, Janusz Kluczyński, Krzysztof Grzelak, Janusz Torzewski, Teresa Fras

**Affiliations:** 1 Institute of Robots & Machine Design, Faculty of Mechanical Engineering, Military University of Technology, 2 Gen. S. Kaliskiego St., 00-908 Warsaw 46, Poland; lucjan.sniezek@wat.edu.pl (L.S.); tomasz.slezak@wat.edu.pl (T.S.); janusz.kluczynski@wat.edu.pl (J.K.); krzysztof.grzelak@wat.edu.pl (K.G.); janusz.torzewski@wat.edu.pl (J.T.); 2 French-German Research Institute of Saint-Louis (ISL), 5 rue du Général Cassagnou, 68301 Saint-Louis, France; teresa.fras@isl.eu

**Keywords:** deformation analysis, residual stresses measurement, AA2519, Ti6Al4V, explosive welding, layered laminate

## Abstract

Explosively welded layered materials made of (a) an AA2519 aluminum alloy (AlCuMgMn + ZrSc), (b) titanium alloy Ti6Al4V and (c) an intermediate layer composed of a thin aluminum alloyed AA1050 layer are considered herein. This study presents test results connected to measurement science including microstructural observations of the material combined with the explosive method, and a basic analysis of the strength properties based on microhardness and tensile tests. Owing to the joint’s special manufacturing conditions, the laminate was subjected to deformation measurements with the digital image correlation (DIC) method. The research was supplemented by the residual stress measurements with the sin2ψ X-ray method based on the diffraction–reflection analysis that was verified by the bore trepanation method.

## 1. Introduction

In this article, a layered composite was tested which will be used as a construction material with increased ballistic resistance for spacecraft and military vehicles. In space technology, the 2519 alloy is used for protective panels against micrometeorites and for the construction of space station chambers [1].

The Ti6Al4V alloy is characterized by increased ballistic resistance and therefore it can be, especially in combination with aluminum alloys with increased ballistic resistance, an alternative to, inter alia, homogeneous armor plates [2,3]. Due to its special functional properties, the Ti6Al4V alloy has long been used in the production of aircraft components, including: jet engine rotor blades and wing caissons, one of the largest elements in the construction of fighters and combat vehicles [4,5]. The Future Combat System (FCS) program developed by the American army assumes that materials that can play a special role in the development of special structures for military purposes, including the construction of combat vehicles, aircraft and satellite structures, include titanium alloy Ti6Al4V, alloys aluminum AA2519 and AA5083 and polymer composites [6,7,8,9]. It is anticipated that the use of the appropriate materials, especially light alloys with different densities and mechanical properties, can provide an appropriate level of strength and ballistic resistance while reducing the weight compared to classic passive shields. The layered structure is to increase the phenomenon of energy dissipation of the hitting projectile and thus increase the ballistic resistance of the material. Traditional joining techniques are not able to effectively join titanium and aluminum alloys, so it was decided to use the explosive joining technique [10,11,12,13].

Combining these theoretically not weldable materials may be by explosive bonding. This process over the years has not been resolved from the theoretical. There are many theories that describe the process of merging, and so far, they have not explained specifically which of them is the most appropriate approach [14,15,16,17,18]. Changing parameter combinations has an amazing effect on the type and quality of the connection. This translates into the subsequent mechanical properties and structural material. There is therefore a need to understand and analytically represent intervention input components affecting the future connection type [19,20,21].

Layered materials are characterized by different mechanical properties than in monolithic parts. This phenomenon was taken into account during our previous research of layered materials obtained through different manufacturing technologies: additively manufactured using selective laser melting technology [22,23,24,25,26], additively manufactured, double-material restraint joints using fused filament fabrication technology [27] and explosively welded lightweight materials [28,29,30]. All the mentioned manufacturing technologies are characterized by the layered structure of the materials obtained by joining each layer of the material using high-energy sources. That kind of approach generates high-level residual stresses which directly affect the mechanical properties of manufactured parts. The joint area is characterized by different mechanical properties to the other part of the material. The mentioned area is very small so to properly verify the residual stresses level, special X-ray technologies have to be used [31].

The produced ballistic material was subjected to basic tests of its strength properties. Metallographic specimens were cut out of the produced material and examined with a scanning microscope. The quality of the connection between the layers of the joined material was assessed. The structure in the joining layers was checked for any separation or fragmentation of the structure. In addition, the chemical composition of selected elements in critical connection points was checked. Due to the innovative joining technique, the last monotonic tensile test of the base materials and the produced laminate were examined. The work also investigates the deformation distribution using the digital image correlation (DIC) method. This test was performed on each side of the laminate produced. During the explosive bonding process, a pressure wave is generated, which imparts very high velocity to the solids (plates made of joined materials). The collision of the joined materials at such high velocities releases pressures of up to 2 × 10^4^ MPa. Such pressure makes it possible to obtain physical states unattainable under static load conditions. The effect of their influence is the presence of significant values of residual stresses, deviating from the primary stress distribution in the joined materials. The type of residual stress has a decisive influence on the functional properties of the material and the level of ballistic resistance. For this reason, the residual stresses will be measured by the X-ray method and the hole-drilling strain gauge method.

## 2. Materials

Composites characterized by the unique properties of their constituent materials often comprise a structural or layered nature. Explosive bonding is a practical-based technique that recognizes the fact that explosive welding is a special type of pressure bonding. Achievement of material connections is possible by the massive impact generated between at least two elements in high-pressure conditions caused by the explosive welding process. This type of technology allows the joining of different types of metals, which cannot be bonded using all types of methods except from gluing or from using conventional mechanical joints. This technology makes the joining of materials which are difficult-to-weld possible, even if these have extremely different properties. The main advantage of this technology is the higher breaking strength of the joints compared to the strengths of the constituent materials of the composite [32]. An additional advantage of this technology refers to the economic benefits that result from the reduction in consumption of expensive materials used as adhesives. All the physical phenomena observed during the explosive bonding process are extremely dynamic. It is thus impossible to analyze and compare phenomena that result from the displacement of joined material sheets within short time periods. This is the main reason according to which it is not pertinent to carry out comparisons of the physical phenomena with more classical welding technologies. Structural changes of joined materials are usually limited only to the connection zone that spans several micrometers. The lack of changes in the material’s chemical composition is justified by the fact that composites retain the unique properties of their constituent materials [33].

**Figure 1:**
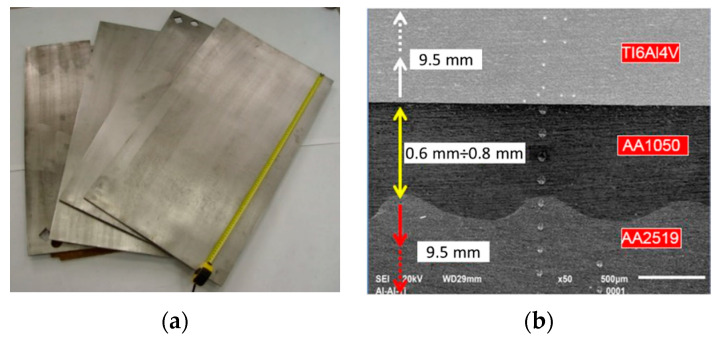
Laminate AA2519-AA1050-Ti6Al4V: (**a**) sheets produced, (**b**) metallographic cross-section of the composite.

The layered composite produced with the use of the explosive welding technology from base materials, i.e., aluminum alloy AA2519 (AlCuMgMn + ZrSc) and titanium alloy Ti6Al4V with the use of an additional intermediate layer of AA1050 alloy with a thickness in the range of 0.6–0.8 mm, was tested. The technological treatment included surface preparation based on surface rolling and grinding. Manufactured laminates are shown in Figure 1a. The cross-section of the laminate shows the connection zone of the Ti6Al4V and AA2519 alloys with the AA1050 alloy interlayer (Figure 1b). On the AA2519–AA1050 border junction, characteristic wavy and flat surfaces were observed in the combined Ti6Al4V–AA1050 construct.

The characteristics of material combinations in terms of wavy structures with similar densities and masses were determined based on the appropriate selection of the joining parameters. The connection quality of a flat border between AA1050 and Ti6Al4V can be demonstrated by the intermetallic precipitations formed in the additional sublayer. For more accurate microstructural analyses, the JEOL JSM-6610 scanning electron microscope (Jeol LTD, Tokyo, Japan) was used. Sample preparations in the form of metallographic samples were required to obtain high-quality surfaces. The classical method of sample preparation (mechanical polishing) could not provide a proper surface quality for all laminate components. Mechanical ion polishing was used only at the initial preparatory stage. Ion polishing is dedicated to sample metallographic testing preparation. The ion-polishing process involved the removal of atomic layers from the material surface with an argon ion beam. The surface preparation of samples for metallographic tests was performed with the Hitachi IM4000 Ion Milling System (Hitachi LTD, Tokyo, Japan) with a protective mask that allowed the generation of smooth surfaces. This technology was dedicated to multicomponent material polishing with constituent materials with different hardness values.

A specially modified AA2519 alloy manufactured at the Non-Ferrous Metals Institute in Gliwice, Poland, was used to produce the composite. Owing to the increased fatigue durability and diminished tendency for fragmentation during the dynamic impact of missiles, the AA2519 alloy was used in the construction of combat vehicles, devices with increased ballistic resistance and in elements dedicated to aerospace applications. In the space industry, AA2519 alloys are used for protective panel construction for devices covering against micrometeorites [34]. The AA2519 alloy modification developed by the Institute of Non-Ferrous Metals consisted of additional alloying elements, including Zr and Sc, in concentrations in the range of 0.2–0.3%. The improvement of the process allows for precipitation strengthening and an increase in the secondary recrystallization resistance of the structure. The mentioned modifications could obtain stable mechanical properties in high-temperature conditions as a result of the homogeneous phase dispersion of Al_3_Zr. The strength properties of AA2519 alloy were obtained on the basis of our own research. The chemical composition of the material was given by the manufacturer. The data are presented in Table 1.

Advantageous mechanical properties, good weldability, high corrosion resistance and a melting point temperature of 1955 K indicate that titanium alloys can be extensively used in the aerospace, space and military industries. The Ti6Al4V alloy is also characterized by increased ballistic resistance that makes it an alternative material to those used for homogeneous armor plate productions [35]. The specific properties of the Ti6Al4V alloy allow its use in the production of aircraft components, including jet engine rotor blades and wing caissons [36]. The strength properties of Ti6Al4V alloy were obtained on the basis of our own research. The chemical composition of the material was given by the manufacturer. The data are presented in Table 2.

The explosive bonding of AA2519 and Ti6Al4V alloys was achieved with the use of an AA1050 aluminum alloy interlayer. The strength properties of AA1050 alloy were obtained on the basis of our own research. The chemical composition of the material was given by the manufacturer. The data are presented in Table 3.

Owing to its high plasticity, the AA1050 alloy has very good adhesive properties which improve the strength properties of the connection zone.

**Figure 2:**
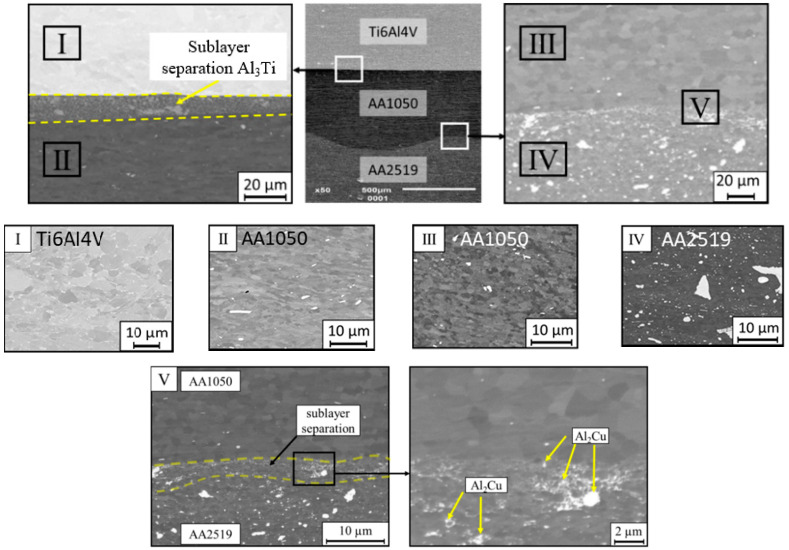
Metallographic cross-sections obtained with scanning electron microscopy of the manufactured laminate AA2519-AA1050-Ti6Al4V.

The microstructure of the AA2519-AA1050-Ti6Al4V joint was considered. Particular attention was paid to areas near the junction zone, such as the area near Ti6Al4V with the AA1050 alloys and that near the AA2919 and AA1050 alloys. The surface of the Ti6Al4V alloy, shown in Figure 2 has a proper structure for this type of material and does not show visible deformations or damages. In the case of the AA1050 aluminum alloy, grain deformations in the form of elongated shapes are visible. This phenomenon is connected to the process of the high pressure generated during the explosive joining process and the location of the material next to the hard deformable titanium alloy. The use of the same material interfaced with the AA2519 aluminum alloy in the connection area retains a regular grain structure. The AA2519 aluminum alloy structure yields fine, copper-rich precipitations, which are visible as bright areas in Figure 2. The presence of copper in the AA2519 aluminum alloy improves the bonding capacity of the explosive.

A more detailed analysis of the AA1050 aluminum joining zone and the AA2519 alloy showed that during the explosive bonding, a precipitation sublayer with an increased copper concentration in phase Θ was formed owing to the increased pressure and short-term temperature growth. This zone is a natural obstacle to the dislocation movement that strengthens the entire alloy. In addition to the increasing distance from the joining zone, grain growth was also observed [37,38].

**Figure 3:**
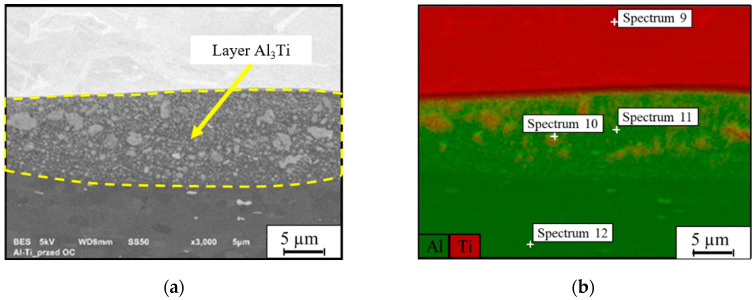
Micro-area of the AA1050-AA2519 material connection zone: (**a**) microstructure, (**b**) chemical composition measurement locations.

In the case of the border of the titanium alloy Ti6Al4V with the 1050 aluminum alloy, an intermediate layer was observed with a thickness of approximately 8–15 µm (Figure 3a). In this layer, the presence of numerous precipitates of various sizes and irregular shapes was found. To identify the chemical composition of materials in the diffusion layer, an energy-dispersive spectrometry (EDS) analysis was performed (Figure 3b).

The results of the additional chemical composition measurements (the spectral points in Figure 3b) indicated the presence of Ti–Al compounds in the intermediate layer. The relative chemical compositions of these elements in the selected spectra are listed in Table 4.

Boronski et al. [39] and Milosavljevi M et al. [40] proved that the observed intermediate layer was formed as a result of aluminum deposition on the titanium substrate due to the high temperature and pressure.

## 3. Testing Basic Strength Properties

**Figure 4:**
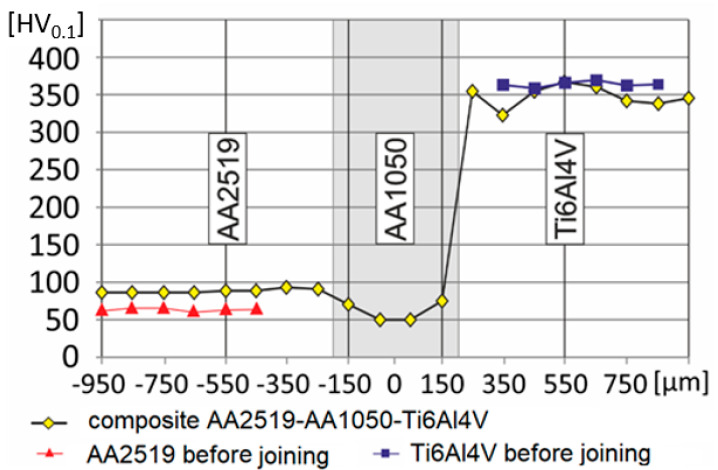
Microhardness distribution in the AA2519-AA1050-Ti6Al4V laminate.

Microhardness measurements were carried out with a Struers DURA SCAN 70 metallographic microhardness tester (Struers Inc, Cleveland, OH, USA) with the Vickers method in accordance with the methodology included in the standard PN-EN ISO 6507-1:2007. The obtained results of the microhardness measurement of the AA2519-AA1050-Ti6Al4V alloys in the connection area, including the microhardness of constituent materials before the connection, are presented in Figure 4.

The gray graphs indicate the transition layer in the form of AA1050 aluminum. The microhardness tests in the laminate indicate an increase in the microhardness of the AA2519 aluminum alloy compared with the base alloy. This change is the effect of the dislocation strengthening phenomenon that results owing to the crumple caused by the explosion.

**Figure 5:**
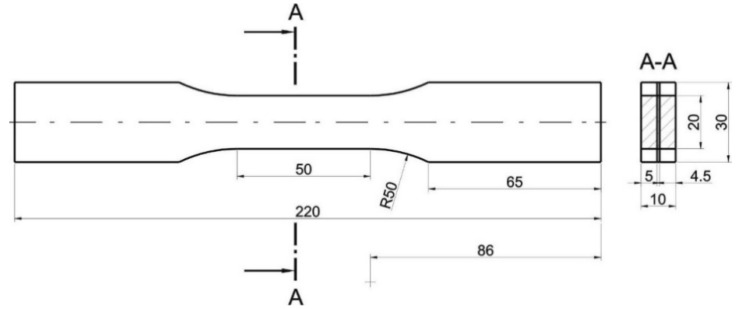
Samples subjected to a static tensile test made of the AA2519-AA1050-Ti6Al4V laminate.

Tensile tests of the AA2519-AA1050-Ti6Al4V laminate and its base materials in the presence of uniaxial, quasi static and crosshead-controlled tensile testing conditions were carried out in accordance with PN-EN ISO 6892-1:2010 with an Instron 8802 hydraulic pulsator (Worldwide Headquarters, Norwood, MA, USA). Deformation measurements were carried out with an Instron 2630-112 extensometer (Worldwide Headquarters, Norwood, MA, USA) with a 50 mm measuring base in the presence of axial stretching conditions. Monotonic tensile tests were carried out on samples made of the manufactured laminate (Figure 5) and on samples produced with the use of base materials before and after heat treatment. All samples subjected to tensile testing had the same geometry. Six samples were made of each material.

**Figure 6:**
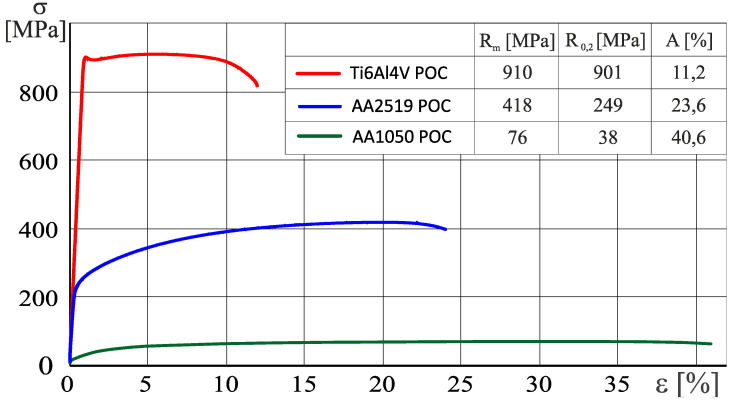
Monotonic tensile test chart for dog-boned samples made of the base material.

The tensile test chart of AA2519-AA1050-Ti6Al4V is shown in the Figure 6. In the case of the titanium alloy, the maximum tensile strength R_m_ = 910 MPa was reached. This value exceeded the maximum tensile strength of the AA2519 alloy (R_m_ = 358 MPa) by >2.5 times. The relative elongation A for the titanium alloy Ti6Al4V is approximately 11.2%, while that for the aluminum alloy AA2519 is approximately 6.8%. The tensile strength R_m_ of the elements produced by the method of explosive welding was 657 MPa, and the conventional yield strength R_0.2_ was 436 MPa, with a total elongation A of 6.5%.

The tensile strength *R_m_* of the manufactured composite in which the constituent materials are characterized by the solid material structure can be compared with the theoretical composite strength calculated based on the “mixtures law”.

For the tested AA2519-AA1050-Ti6Al4V material, the mixtures law can be expressed as

*R_mk_ = R_mw_ × V_w_ + R_mo_ × V_o_+ R_mm_ × V_m_* (1)

where *R_mk_* is the composite tensile strength, *R_mw_* is the tensile strength of the base material, *V_w_* is the tensile strength ratio of the reinforcement cross-sectional area to the matrix cross-sectional area, *R_mo_* is the warp tensile strength, *V_o_* is a ratio of the matrix cross-sectional area to the strengthening cross-sectional area, *R_mm_* is the interlayer resistance and *V_m_* is the ratio of the cross-sectional area of the interlayer to the cross-sectional area of reinforcement.

**Figure 7:**
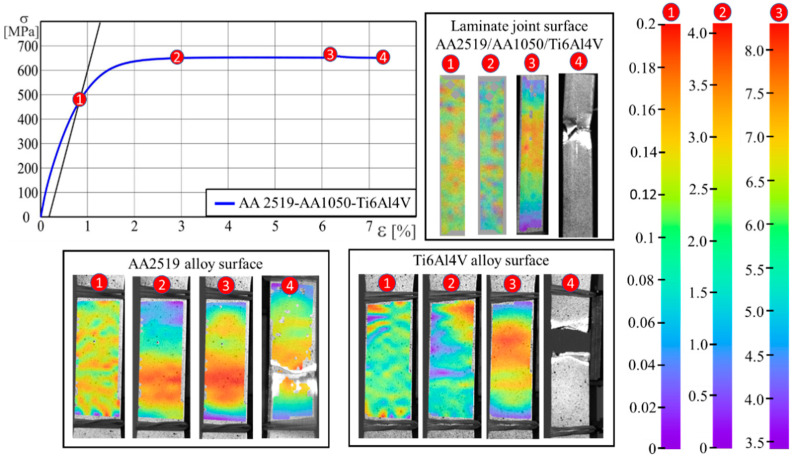
Deformation distribution using the digital image correlation (DIC) method for selected points of the monotonic tensile test of a flat-layered AA2519-AA1050-Ti6Al4V composite sample: 1: conventional yield point *R*_0.2_, 2: tensile strength for ε_p_ = 2%, 3: maximum tensile strength *R_m_* and 4: crack sample.

The theoretical tensile strength was determined on the basis of the mixtures law for *R_mk_* = 604 MPa. The obtained test results carried out on the produced laminates indicate that the tensile strength of the AA2519-AA1050-Ti6Al4V laminate is *R_m_* = 657 MPa (Figure 7). Explosive bonding increased its strength by >8%. The observed increase in the *R_m_* value was influenced by the strengthening of the constituent material caused by the crushing during the evolution of the explosive bonding process. Another factor that increases tensile strength is the formation of Al_3_Ti intermetallic phase materials in the intermediate layer [41,42]. Owing to the diffusive nature of the Al-Ti joint formation process and the thermo-mechanical processes, elements are segregated in a very narrow area of the transition zone. This results in a local change in mechanical properties. These factors in conjunction with the layered structure of the produced composite can have a significant impact on the different values of the material deformation. Verification of the laminate surface deformation process in a monotonic tensile test was carried out with the use of the digital image correlation (DIC) method. Deformation observations were carried out from the three sides of the laminate, namely the aluminum alloy, titanium alloy and side sample surfaces. The tests results are shown in Figure 7.

Obtained test results for points 1 and 2 (in Figure 7) yield homogeneous strain distributions on all sides. Visible material deformation areas were only observed after the yield strength was exceeded. It should be noted that the images of deformation fluctuations on the side of the sample did not yield banding effects. This may be attributed to the layered arrangement of materials with different strength properties. At point 3 in Figure 7, the concentration of maximum deformations was observed at the crack initiation site. The location of the maximum deformations and the continuous nature of their growth indicate joint plain consistency within the entire range of the load during the test. Sample cracks were present in the zones of maximal deformation.

## 4. Residual Stress Measurements

### 4.1. X-ray Stress Measurements

**Figure 8:**
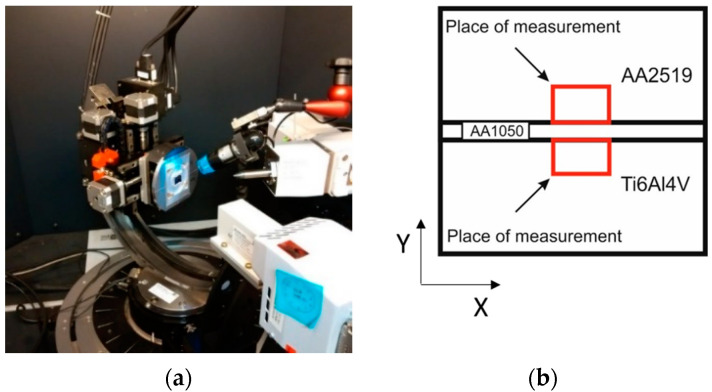
Bruker D8 Discover diffractometer with a Euler wheel (**a**), and locations of areas of self-stress measurements based on the X-ray method (**b**).

X-ray stress tests were carried out in cooperation with the Institute of Metallurgy and Materials Engineering of the Polish Academy of Sciences in Krakow. The tests were conducted on a Bruker D8 Discover X-ray diffractometer (Bruker AXS GmbH, Karlsruhe, Germany) with a Euler wheel (Bruker AXS GmbH, Karlsruhe, Germany) and a sample positioning table (Figure 8).

**Figure 9:**
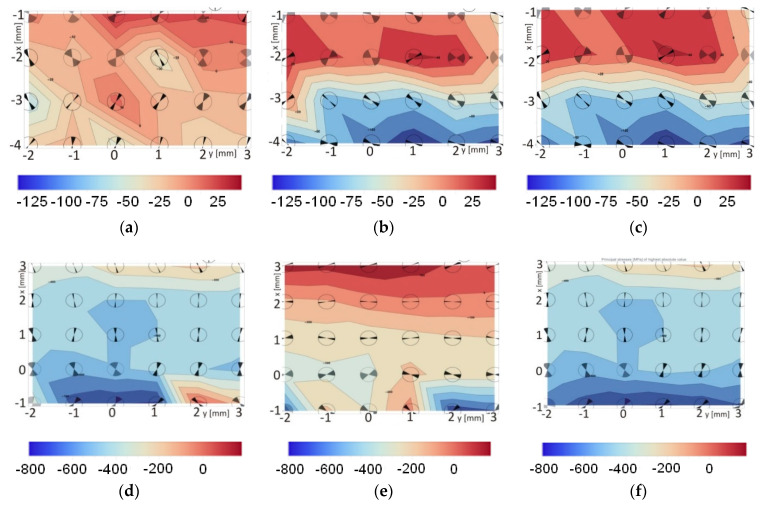
Distribution of residual stress in a sample made of the AA2519-AA1050-Ti6Al4V laminate after explosive joining: AA2519 stresses in vertical direction (**a**), AA2519 stresses in the horizontal direction (**b**), AA2519 principal stresses (**c**), Ti6Al4V stresses in vertical direction (**d**), Ti6Al4V stresses in the horizontal direction (**e**), Ti6Al4V principal stresses (**f**),

X-ray stress measurements were carried out on the surface of the cross-sections of the sample. Measurements were made locally at various points (separated by 1 mm distances) on the surface (area of 25 mm^2^) in accordance with Figure 9. During the tests, standard residual stress measurements were made according to the sin2ψ method using a scintillation detector in a parallel beam system with a Soller 0.34 collimator. The Texture-aided Residual Stress Investigation System software package (TARSIuS) was developed by Professor Bonarski and Mr. Kania from the Institute of Metallurgy and Materials Science of the Polish Academy of Sciences. The software regulates the analysis process of the tested samples, including the application of direct control of the diffractometer during measurements, or the automatic processing of partially acquired results and the visualization of the final results in the form of stress maps. The identified residual stresses in the tested samples are compressive and tensile. To facilitate the interpretation of the stress values, two-dimensional maps are presented separately in the graphical form of the tested composites’ measurements (Figure 9). Measurements for the AA2519 alloy are shown in Figure 9a–c, and for the Ti6Al4V alloy in Figure 9d–f.

**Figure 10:**
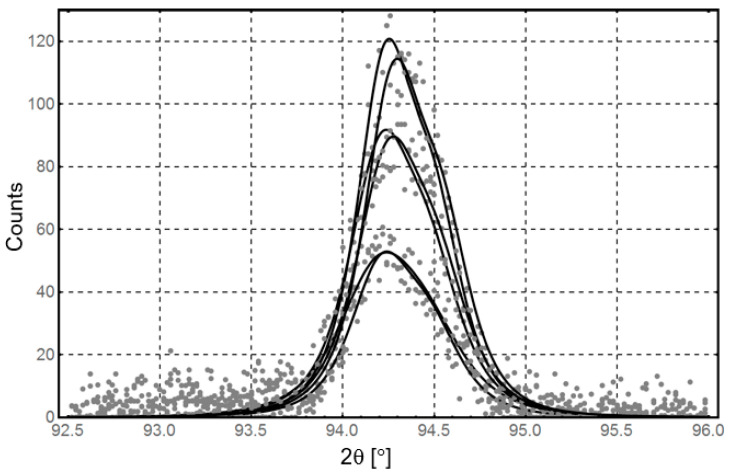
Distribution of intermediate results of one measuring direction for AA2519 alloy.

In the composite formed after explosive welding, the topographies of the main stresses in the titanium and aluminum layers are characterized by their laminar natures that are determined by the interaction of both composite component layers. The titanium layer is characterized by considerably increased stress values (up to −800 MPa) compared with the aluminum layer (up to −100 MPa). The closer the titanium layer is to the intermediate layer, the more profound is the increase in the compressive stress. Near the AA1050 alloy layer, compressive stresses are transformed into tensile owing to the shear forces that increase the effect generated by the crystal lattice mismatches in the two joined materials. The intermediate results obtained for one of the directional stress measurements in this alloy are illustrated in Figure 10. The recorded peaks differ significantly in intensity, and the obtained *d_hkl_* relations are neither linear nor elliptical.

Reproduction of reliable curves based on the obtained measurement data using the Reuss methodology. In these cases, reliable interpretation of stress measurements was not possible owing to the grain growth and/or the presence of precipitations.

### 4.2. Residual Stresses Based on the Hole-Drilling Strain Gauge Method

**Figure 11:**
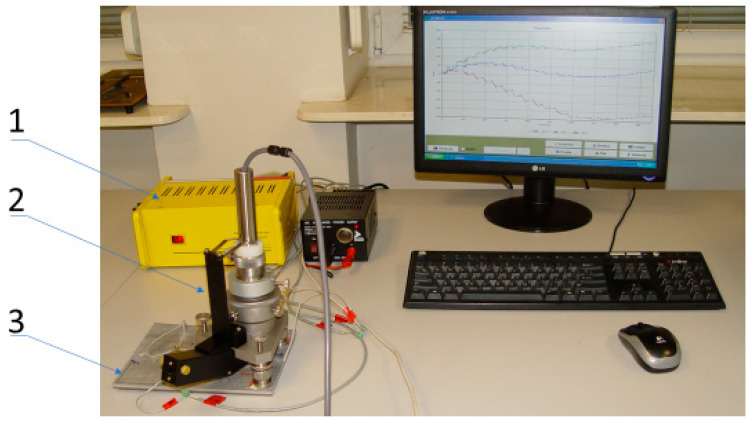
Stand for residual stress measurements: Esam Traveler Plus strain gauge bridge with a power supply (1), RS-200 device (2) and tested material (3).

Nondestructive methods used to measure stress are subject to increased uncertainties. It is advisable to carry out measurement verification using experimental methods. One of the most commonly used methods for this type of experiment is bore trepanation using standard strain gauges. This method is often referred to as the “semidestructive technique” because the hole does not usually cause significant damage to the structural integrity of the test object (usually holes have a diameter of 0.8 mm and a depth of 4.8 mm). Removing material using a drill allows measurements of deformation changes caused by the relaxation of residual stress. In this research study, a hole-cutting device was used to perform hole trepanation based on the process described in the RS-200 Milling Guide-VPGMicro-Measurements. This measurement system (Figure 11) was adapted to allow the precise drilling of holes at the marked points of the geometric rosette at specific depths with simultaneous measurement of its diameter.

**Figure 12:**
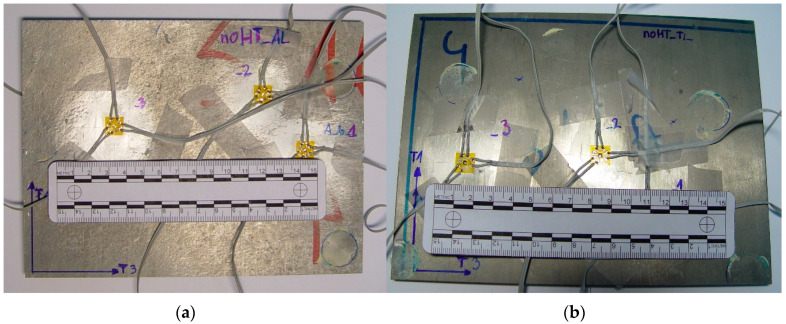
Emplacement of measuring rosettes on a section of the AA2519-Ti6Al4V laminate plate in the initial state: on the aluminum alloy side (**a**), on the titanium alloy side (**b**).

Residual stress measurements were carried out on the produced laminate from the titanium and aluminum alloy sides. The preparation of the surface before the installation of the strain gauge rosettes was carried out based on the guidelines contained in the Instruction Bulletin B-129-8 Surface Preparation for Strain Gauge Bonding, which included the degreasing of the aluminum and titanium surfaces and grinding. The prepared surfaces were stripped of oxides with Conditioner A, and were then neutralized with Neutralizer 5A, which allowed the application of strain gauges. The measurement methodology has been developed for the ASTM Standard Test Methods E 837. It included the attachment of at least three strain gauges with cable installations on each type of tested surface. The EA-13-062RE-120 rosettes were used for the aluminum alloy, while the EA-05-062RE-120 rosettes were used for the titanium alloy. The next step was to drill holes (with a diameter of 1.6 mm and a maximum depth of 2.0 mm) in designated measurement locations at 0.1 mm increments. Voltage signals from strain gauges were amplified by means of the Esam Traveler Plus-type 1032-S Strain Gauge Bridge. Residual stresses and their angular orientations were determined on the basis of measured strain values. Figure 12 shows the emplacement of strain gauges on plates of the laminate produced for the needs of conducting tests using the hole trepanation method.

The resulting output signal registered in the respective depths of the bore was then converted to strain values in accordance with Equation (2).

(2) $$\varepsilon =\frac{4\cdot U_{WY}}{N\times U_{0}\times K\times A}\times 10^{6}\;\left[\mu \varepsilon \right]$$

where *U_WY_* is the output voltage [V], *U*_0_ is the input voltage [V], *N* is the bridge layout factor for the quarter bridge *N* = 1, *K* is the strain gauge constant and *A* is the output signal amplification.

As a result of the measurements and calculations, the characteristics of the relative deformation changes as a function of the depth of the hole were obtained. To determine the values of the main stresses and their direction, the following dependencies were used:

(3) $$\sigma_{max}=\frac{\varepsilon_{1}+\varepsilon_{2}}{4\times A}-\frac{1}{4\times B}\sqrt{{\left(\varepsilon_{3}-\varepsilon_{1}\right)}^{2}+{\left(\varepsilon_{3}+\varepsilon_{1}-2\varepsilon_{2}\right)}^{2}\;}\left[MPa\right]$$

(4) $$\sigma_{min}=\frac{\varepsilon_{1}+\varepsilon_{2}}{4\times A}+\frac{1}{4\times B}\sqrt{{\left(\varepsilon_{3}-\varepsilon_{1}\right)}^{2}+{\left(\varepsilon_{3}+\varepsilon_{1}-2\varepsilon_{2}\right)}^{2}\;}\left[MPa\right]$$

(5) $$\alpha =\frac{1}{2}arctg\frac{\varepsilon_{1}-2\varepsilon_{2}+\varepsilon_{2}}{\varepsilon_{2}-\varepsilon_{1}}\left[MPa\right]$$

where *σ_max_* and *σ_min_* are the principal stresses, *ε*_1_, *ε*_2_ and *ε*_3_ are the strains measured on strain gauges 1, 2 and 3 and *A* and *B* are the coefficients that depend on the material properties, measuring rosettes and hole geometry, while *α* is the angle between strain gauge 1 and the direction of the nearest residual stress.

**Figure 13:**
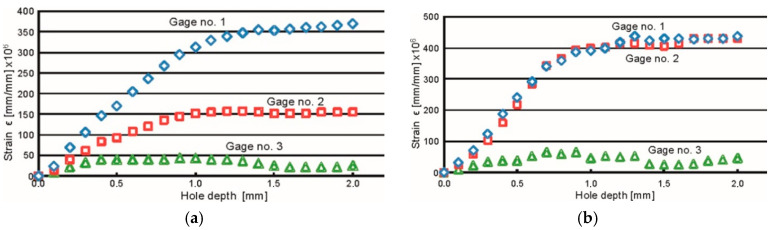
Graphs of strain changes depending on the hole depth on the AA2519 side (**a**) and on the Ti6Al4V alloy side (**b**) of the AA2519-AA1050-Ti6Al4V laminate.

The results obtained for selected measuring points are illustrated in the form of strain change courses as a function of the hole depth. Figure 13 shows the results for the AA2519-AA1050-Ti6Al4V laminate.

The minimum values of principal residual stress *σ_min_* determined at all the measurement points in the case of the AA2519 alloy differ from each other (in the range of −121 to −104 MPa), while the maximum values of *σ_max_* differences are in the range of −88 to −31 MPa. The observed differences in the values of *σ_min_* and *σ_max_* are associated with stress value changes as a function of distance from the material surface. The orientation analysis of the principal stress vector showed that the direction of the minimum stress *σ_min_* was almost perpendicular with respect to the longer edge of the plate, while the maximum stress direction *σ_max_* was parallel to this edge. The residual stress level in the aluminum alloy was very close to a homogeneous distribution (invariant as a function of depth). No tensile stresses were recorded. The stress values determined at all the measured points in the Ti6Al4V alloy were in the following ranges: *σ_min_* = −349–−184 MPa and *σ_max_* = −111–−140 MPa. The obtained test results are associated with changes in stress values as a function of the measurement depth. The analysis of the residual stress orientation vector showed that the direction of the external load that affected the tested laminate was perpendicular to the longer edge of the plate. This caused higher compressive stresses that were similar to those observed during the measurements of the internal stress in the aluminum alloy AA2519.

## 5. Conclusions

Explosive bonding enables the combination of hard bond materials, such as titanium and aluminum alloys. The combination of the proposed method generated an interlayer between the base materials in the microstructure. As a result of the short-term effects of the high pressure in the layer between the Ti6Al4V and AA1050 alloys, Al_3_Ti precipitates were formed. This interlayer significantly changed the functional properties of the material. It was used to form ballistic panels in spaceships. Recognition of the stress values will allow for improving the numerical design in the phenomena of ballistic resistance [43]. In addition, the explosive joining process increased the tensile strength by approximately 8% compared with the strength that resulted from the law of mixtures. Measurements of internal stress by both X-ray and bore trepanation methods showed an increase in the internal stress near the joining zones. Long-range testers should consider the heat treatment of the laminate to relax natural stress.

It should be noted that the stress values obtained in the titanium alloy may be subject to increased uncertainty. This is owing to the fact that titanium cannot be easily cut. Titanium tends to cause increased temperatures in the vicinity of the hole surface owing to the increased friction between the drill and the low-thermal conductivity titanium surface. The described difficulties caused an increase in the cutting resistance and interfered with the recorded output signal that manifested in the form of changes in voltage pulses toward negative values, i.e., caused tensile stresses. These assumptions confirmed that there was a significant increase in the cutting resistance that caused either a decrease in the rotational speed of the tool, or stopped the drill completely, followed by an abrupt increase in the output voltage. It should be stated that certain stress values in the titanium alloy may be altered, however, the degree of this disturbance is impossible to determine.

## Figures and Tables

**Table 1:** Strength properties and chemical composition of the AA2519 alloy.

Strength Properties			Chemical Composition [wt %]									
R_0.2_ [MPa]	R_m_ [MPa]	A [%]	Si	Fe	Cu	Mg	Zn	Ti	V	Zr	Sc	Al
312	335	6.5	0.06	0.08	5.77	0.18	0.01	0.04	0.12	0.2	0.36	rest

**Table 2:** Strength properties and chemical composition of the Ti6Al4V alloy.

Strength Properties			Chemical Composition [wt %]							
R_0.2_ [MPa]	R_m_ [MPa]	A [%]	O	V	Al	Fe	H	C	N	Ti
950	1020	14	<0.20	3.5	5.5	<0.30	<0.0015	<0.08	<0.05	rest

**Table 3:** Strength properties and chemical composition of the AA1050 alloy.

Strength Properties			Chemical Composition [wt %]							
R_0.2_ [MPa]	R_m_ [MPa]	A [%]	Fe	Si	Zn	Mg	Ti	Mn	Cu	Al
78	168	2.9	0.4%	0.25<	0.07<	0.18	0.05<	0.05<	0.05<	rest

**Table 4:** Results of chemical composition measurements in the connection zone of the Ti6Al4V and AA1050 aluminum alloys.

Measurement Point Name	Chemical Composition [%]	
	Ti	Al
Spectrum 9	92.8	7.2
Spectrum 10	69.6	30.4
Spectrum 11	83.1	16.9
Spectrum 12	0	100
